# Piezo1 in vascular remodeling of atherosclerosis and pulmonary arterial hypertension: A potential therapeutic target

**DOI:** 10.3389/fcvm.2022.1021540

**Published:** 2022-09-29

**Authors:** Han Xu, Yu He, Tianying Hong, Cong Bi, Jing Li, Mingfeng Xia

**Affiliations:** ^1^Innovation Research Institute of Traditional Chinese Medicine, Shandong University of Traditional Chinese Medicine, Jinan, China; ^2^Cardiovascular Surgery Department, The First Affiliated Hospital of Xi'an Jiaotong University, Xian, China; ^3^Department of Vascular Surgery, Shandong Provincial Hospital Affiliated to Shandong First Medical University, Jinan, China

**Keywords:** vascular remodeling, mechanosensitive ion channel Piezo1, endothelial cells, vascular smooth muscle cell, pathology

## Abstract

Vascular remodeling (VR) is a structural and functional change of blood vessels to adapt to the changes of internal and external environment. It is one of the common pathological features of many vascular proliferative diseases. The process of VR is mainly manifested in the changes of vascular wall structure and function, including intimal hyperplasia, thickening or thinning of media, fibrosis of adventitia, etc. These changes are also the pathological basis of aging and various cardiovascular diseases. Mechanical force is the basis of cardiovascular biomechanics, and the newly discovered mechanical sensitive ion channel Piezo1 is widely distributed in the whole cardiovascular system. Studies have confirmed that Piezo1, a mechanically sensitive ion channel, plays an important role in cardiovascular remodeling diseases. This article reviews the molecular mechanism of Piezo1 in atherosclerosis, hypertension and pulmonary hypertension, in order to provide a theoretical basis for the further study of vascular remodeling.

## Introduction

Vascular remodeling (VR) is a frontier field in the research of various cardiovascular diseases in recent years. As a dynamic pathological process, it is the change of the structure and function of blood vessels to adapt to the changes in the internal and external environment (1–3). The functional changes are manifested as alterations in vascular compliance and derangements in vascular regulation, while the structural changes are reflected in the synthesis, degradation and reorganization of the extracellular matrix (ECM) and the excessive proliferation, migration and apoptosis of vascular endothelial cells and smooth muscle cells (2, 4–8). VR is a key pathological characteristics of the development of a variety of cardiovascular diseases, and many biological processes can lead to pathological VR, such as endothelial cell dysfunction and damage, vascular smooth muscle cell migration, proliferation, apoptosis, oxidative stress, lipid accumulation, inflammatory response and imbalance of ECM synthesis and degradation (9–13). In addition, VR is also regulated by vascular growth factors, the vasoactive substances and hemodynamics (1, 14, 15). The molecular mechanisms of action of pathological VR are not well understood, which makes the treatment of VR disorders difficult and the prognosis poor (12). Therefore, exploring the pathogenesis and treatment strategies of VR disorders has become a hot topic in basic research, and it is also an urgent medical problem to be solved in clinical work.

The mechanosensitive ion channel Piezo1 is a mechanically activated, non-selective cation channel that efficiently converts mechanical forces into electrochemical signals (16). Piezo1 has been studied in a variety of disciplines, including biology, pathology and pathophysiology, and is manifested in a variety of biological functions such as touch, proprioception, pain, vascular development and blood pressure regulation (17–20). It has been established that Piezo1 is expressed on vascular endothelial cells, blood cells, epithelial cells and cardiomyocytes, etc and plays an important regulatory role in the physiopathological processes of the body (21, 22). Piezo1 acts in the cardiovascular system mainly by sensing the fluid shear stress caused by blood flow and non-selectively mediating the entry of cations such as Na^+^, Ca^2+^ into the cells (23, 24). Piezo1-mediated Ca^2+^ inward flow in vascular endothelial cells can affect vascular remodeling by regulating downstream signaling pathways related to vascular tension, vascular development, lymphatic valve formation and epithelial cell homeostasis, among other effects (25–30). Therefore, the present paper investigates the effects of Piezo1 on vascular remodeling. Thus, the present paper reviews the mechanosensitive ion channel Piezo1 in cardiovascular remodeling diseases, with the aim of providing a theoretical basis for the diagnosis and treatment of cardiovascular remodeling diseases.

## Piezo1 channels structure-function

Piezo1 protein, first reported in 2006, is a Ca^2+^ ion channel membrane protein (31). At the beginning of 2010, Patapoutian's team first screened Neuron2A cell lines to determine that they could sense mechanical stress, and then used modern molecular biology techniques such as gene silencing and membrane clamp to silence 72 candidate genes with RNA, followed by stress testing and recording of currents in the treated cells (32). Coste et al. (32) eventually identified Fam38A, the gene that mediates mechanosensitive ionic currents, and found that knocking out the Fam38A gene eliminated currents activated by mechanical forces, naming it Piezo1, a discovery that was also awarded the Nobel Prize in Physiology or Medicine in 2021.

The Piezo1 protein contains 2,547 amino acids. It is a trimeric propeller-shaped channel protein (~900 kDa), consisting of a central anchor, three long beams and three blade-like structures (33). Observed under high-resolution cryo-electron microscopy (cryo-EM), it shows a three-leafed helical structural state (33–35). It is the unique structure of Piezo1 that allows it to respond with very specific mechanosensitivity to external stimuli and to internal signals generated by the cell (36).

It was demonstrated that Piezo1 can sense and transmit a variety of mechanical forces, including cell membrane tension (37), shear stress (17, 38), cellular stretch (32), and cyclic pressure (39). Increasingly, Piezo1 has been confirmed to play an important role in the maintenance of vascular development and vascular function, particularly in the regulation of vascular endothelial cell function (40). It was found that Piezo1 is highly expressed on vascular endothelial cells, and when researchers knocked out Piezo1 on mouse endothelial cells, it was observed that blood vessels failed to form in mouse embryos, resulting in embryonic death; experiments confirmed that Piezo1 has an important role in vascular development and molding (17, 41, 42). In addition, Retailleau et al. (43) found that in arterial smooth muscle cells, Piezo1 induced small arterial vascular neovascularization by activating TG and promoting thickening of small arterial blood vessel walls.

It has also been reported that vascular endothelial cells constantly interact with the intra- and extra-luminal extracellular environment and that endothelial cells are endowed with physical stresses, such as shear stresses and tensile forces, which are sensed and transmitted by Piezo1, leading to altered cell behavior with excessive proliferation, migration and aggregation, forming primitive vascular vasculature and further leading to pathological vascular remodeling (28, 44–46). In summary, Piezo1 has a major function in vascular remodeling and may be a potential therapeutic target for vascular remodeling diseases.

## Pharmacological modulators of Piezo1 channels

### Piezo1 channel activator

In addition to physical-mechanical forces, Piezo1 also allows gating by chemical means. Syeda et al. (47) recently used high-throughput screening techniques to screen 3.25 million low molecular compounds one by one, eventually identifying the first chemical activator of the Piezo1 channel and naming it Yoda1, which activates Piezo1 without mechanical stimulation by acting on the intracellular region at the C-terminus of the Piezo1 channel protein (48–51). The results of the experimental research showed that the activation current of Piezo1 channels was found to be significantly reduced after depletion of extracellular Ca^2+^ with Ca^2+^ chelators, but was not altered when intracellular Ca^2+^ was depleted by the application of toxic carotene (Thapsigargin, A potent inhibitor of intracellular calcium transport enzymes), indicating that the Yoda1-induced Ca^2+^ response is likely to be dependent on the inward flow of extracellular Ca^2+^. In addition, it was found that the administration of certain mechanical stimuli enhanced the kinetic process of Yoda1-induced Piezo1 channels and prolonged the inactivation of transient currents (36, 49, 52, 53).

Jedi1/2 is a new recently discovered hydrophilic Piezo1 channel chemical activator that acts by acting on a distal paddle at the cell periphery, using a peripheral lever-like device consisting of a blade and a light beam to control the central Piezo1 ion conductance pore (54, 55). However, the specific mechanisms by which these chemicals activate Piezo1 channels remain elusive, and their role in cardiovascular remodeling needs to be further investigated.

### Piezo1 channel non-specific inhibitor

Only a few drugs have been used in pharmacological studies of Piezo1 channel Non-specific inhibitors, broadly classified as Gd3^+^, Dooku1, and La3^+^ in the lanthanide family, gentamicin and streptomycin in the aminoglycoside group, ruthenium red (RR) and the spider venom peptide GsMTx-4 isolated from tarantula toxin (16, 32, 56–60). GsMTx-4 is a cation channel-specific blocker that not only blocks Piezo1 ion channels but also has the effect of inhibiting Piezo1-induced mechanically activated (MA) current generation (61–64). Mechanistically, GsMTx4 binds to the cell membrane and, by inserting its hydrophobic domain into the lipid bilayer, acts directly on the backbone proteins of the membrane, blocking mechanical force transmission by altering the tension in the membrane and thus achieving a blockade of the Piezo1 ion channel (62, 65, 66). It has been confirmed that two drugs, such as Phosphatidic acid (PA) and lysophosphatidylcholine (LPC), also exert a blocking effect on the mechanical ion channel Piezo1 *via* the above pathway (20, 66–69).

Previous research in the author's laboratory has revealed that the effective extract of the Chinese medicine tubeimoside I (TBMS1) antagonizes the activation of Piezo1 channels by Yoda1 through competing with Yoda1 for the target of action, thereby inhibiting the Yoda1-induced aortic diastole. Meanwhile, it was also found that although TBMS1 did not significantly inhibit phenylephrine-induced aortic ring contraction, it significantly reduced Yoda1-induced vasodilation, suggesting that TBMS1 directly inhibits Piezo1 channel activity or functions through other unknown mechanisms, thus speculating that TBMS1 may act on Piezo1 in vascular smooth muscle cells to partially inhibit vasoconstriction and thus slow down the onset of vascular remodeling (50). The above findings also confirm that herbal compounds can act through Piezo1 ion channels for the treatment of vascular remodeling diseases, which may also provide new targets for the development of disease-targeting drugs (Table 1).

**Table 1:** Summarization of current Piezo1 pharmacology.

**Category**	**Drugs**	**Selectivity**	**Binding site (domain)**	**References**
Activating agents	Yoda1	Selective	C-terminal (ATM area)	(36, 48–52)
	Jedi1/2		L15-16/L19-20 area	
Inhibiting agents	GsMTx4	Non-selective	Pore of the channel	(53, 56–59)
	Ruthenium red (RR)			(32, 54)
	Gd3^+^			(54, 55)
	Tubeimoside 1		Compete with Yoda1	(48, 60)
	Dooku1			
Modulating agents	lysophosphatidylcholine (LPC)	Non-selective	Membrane lipid environmental alterations	(20, 59–62)
	Phosphatidic acid			

### The role of Piezo1 in atherosclerotic vascular remodeling

Atherosclerosis (AS) is the most common type of atherosclerosis, which is characterized by the accumulation of lipids and necrotic tissue in the intima of arteries, forming yellow atheromatous plaques (61, 70). AS is a chronic inflammatory disease and the pathological basis of many cardiovascular diseases. As the disease progresses, the lumen gradually becomes thickened, stiffened, and less elastic until it becomes occluded, eventually leading to myocardial infarction, stroke and other diseases (71–73).

It is well known that inflammation is a key factor driving the development and progression of AS, and that disturbed blood flow shear stress can exacerbate the inflammatory response by damaging vascular endothelial cells (28, 74–76). Albarran-Juarez et al. (77) demonstrated that Piezo1 activates P2Y2 receptors and Gq/G11 protein-mediated integrins after sensing vascular perturbations, which in turn activates adhesion kinase-dependent activation of the pro-inflammatory star factor NF-κB, thereby exacerbating the development of atherosclerosis. It was also found that when endothelial-specific Piezo1 or Gq/G11-deficient mice were induced, activation of integrins, activation of inflammatory signaling pathways was found to be reduced, and the area of atherosclerosis and the extent of atherosclerosis were both reduced to some extent. Further studies revealed that under high shear stress laminar flow, Piezo1-mediated vascular remodeling-related signaling pathways P2Y2 and Gq/G11 were inhibited, but activated downstream eNOS signaling pathways, which were instead protective against atherosclerotic disease (77–80). Experiments have shown that Piezo1 plays a regulatory role in the development of atherosclerotic pathology by mediating different downstream signaling pathways through sensing different blood flow patterns such as laminar and perturbed flow.

Proliferation, apoptosis and migration of vascular smooth muscle cells are important pathological changes in diseases such as atherosclerosis and restenosis and are major pathological features of arterial reconstruction due to mechanical stress (81, 82). Piezo1, as a mechanosensitive ion channel protein, is essential for the sensing and transduction of mechanical forces such as shear stress and tensile force (83, 84). Randolph et al. (41, 85, 86) showed that Piezo1 mediates cell proliferation-related factors such as matrix metalloproteinase-2 (MMP-2), matrix metalloproteinase-9 (MMP-9), and Platelet derived growth factor (PDGF) expression directly mediates the proliferation of vascular smooth muscle cells. In addition, the application of a 15% mechanical stretching force to mouse aortic smooth muscle cells in cell culture using the Flexcell cellular fluid shear stress system revealed that the expression of proteins and genes related to the Akt signaling pathway, which regulates cell proliferation, migration and anti-apoptosis, was upregulated, leading to sustained smooth muscle cell proliferation and exacerbating the progression of atherosclerotic disease (87–91). Meanwhile Jufri et al. (92) suggested that physiological mechanical stretch helps maintain vascular health and that pathological stretch leads to further disease development. When endothelial cells were stretched by pathological mechanical forces, elevated expression of specific vascular smooth muscle cell marker genes (SM22, SMA, Caldesmon-1, SM MHC and Calponin) and decreased expression of endothelial markers were observed, suggesting that endothelial cells are gradually damaged after a certain mechanical stretch force and smooth muscle cells proliferate abnormally, which is also driving plaque This is one of the important reasons for the progression of plaque (93–95).

This suggests that inhibition of Piezo1 overexpression may have a therapeutic effect on atherosclerotic disease. Recent studies in the author's laboratory have found that silencing the expression of Piezo1 in human umbilical vein endothelial cells (HUVEC) and murine liver endothelial cells (MLEC) has a protective effect against atherosclerosis. It was also demonstrated that the Chinese herbal medicine monomer salvianolic acid B could inhibit Ca^2+^ influx following Piezo1 activation by Yoda1, which inhibited pathological aortic luminal hypoelasticity and thus slowed down the formation of atherosclerotic plaques (96).

### The role of Piezo1 in vascular remodeling in pulmonary hypertension

Pulmonary arterial hypertension (PAH) is a complex, progressive cardiopulmonary disease characterized by non-specific symptoms such as dyspnoea, fatigue, weakness, angina, and syncope. The main features of PAH are increased pulmonary vascular resistance and elevated pulmonary arterial pressure, ultimately leading to right heart failure and death (97–99). It has been shown that the main factors contributing to increased pulmonary vascular resistance during the development of PAH are abnormal systolic function of the small pulmonary arteries and remodeling of the pulmonary arteries (100–102). An increase in cytoplasmic free Ca^2+^ concentration ([Ca^2+^] cyt) in pulmonary arterial smooth muscle cells (PASMCs) is the main cause of pulmonary vasoconstriction, while an imbalance in intracellular calcium (Ca^2+^) homeostasis stimulates PASMCs to proliferate and inhibits their apoptosis, leading to pulmonary vascular remodeling. The imbalance in intracellular calcium (Ca^2+^) homeostasis stimulates PASMCs to proliferate and inhibits their apoptosis, leading to pulmonary vascular remodeling and driving PAH disease progression (53, 101–105). Under physiological conditions, pulmonary endothelial cells Piezo1 regulate endothelium-dependent pulmonary vasodilation by mediating Ca^2+^ inward flow (83, 105–107). It has been hypothesized that upregulation of Piezo1 expression in pulmonary arterial endothelial cells (PAECs) and pulmonary artery smooth muscle cells may play a key role in vascular remodeling in PAH disease. Piezo1 high expression induced Ca^2+^ influx in PAECs, and upregulated Notch ligands (JAG-1, and DLL4) in PAECs. The increased Notch ligands in PAECs, as signal-sending cells, then activate Notch receptors in PASMCs, as signal-receiving cells, and result in pulmonary arteriole muscularization and concentric pulmonary vascular remodeling (105). This suggests that Piezo1 plays a key role in regulating endothelial cell Ca^2+^ homeostasis and is important for pulmonary artery vascular remodeling.

Pulmonary vasodilation is closely related to the regulatory role of PAECs, but the biological mechanism of Piezo1 in PASMCs is unclear. It has been proposed that alterations in hemodynamics cause endothelial damage in the pulmonary vasculature to some extent and also play a regulatory role in the phenotypic transformation and morphological structure of PASMCs, and that hemodynamics are also necessary for the development and maintenance of vascular remodeling in pulmonary hypertension (105, 108, 109). It has been hypothesized that one of the important factors contributing to pulmonary artery remodeling is the increase in intracellular free calcium concentration ([Ca^2+^] i) caused by mechanical stimulation of PASMCs (93, 94). Experimental studies have shown that under pulmonary hypertension, elevated [Ca^2+^] i in PASMCs occurs, which also upregulates the expression of genes activating Ca^2+^-sensitive transcription factors such as STIM2, TRPC6 and Orai2, thereby differentiating the phenotype of PASMCs from a contractile/resting phenotype to a proliferative/synthetic phenotype, leading to vascular remodeling (94, 110, 111). In addition, it has been proposed that Piezo1 activates Ca^2+^-dependent signal transduction pathways such as YAP and other related transcription factors following an increase in intracellular cytoplasmic free calcium concentration, resulting in increased mechanosensitivity of the pulmonary vasculature to regulate cell proliferation and pulmonary vascular remodeling, and that aberrant mechanical stimulation of Piezo1 in PASMCs exacerbates blood flow disturbances, a process that circumferentially also promotes progression of pulmonary hypertension (53).

**Figure 1:**
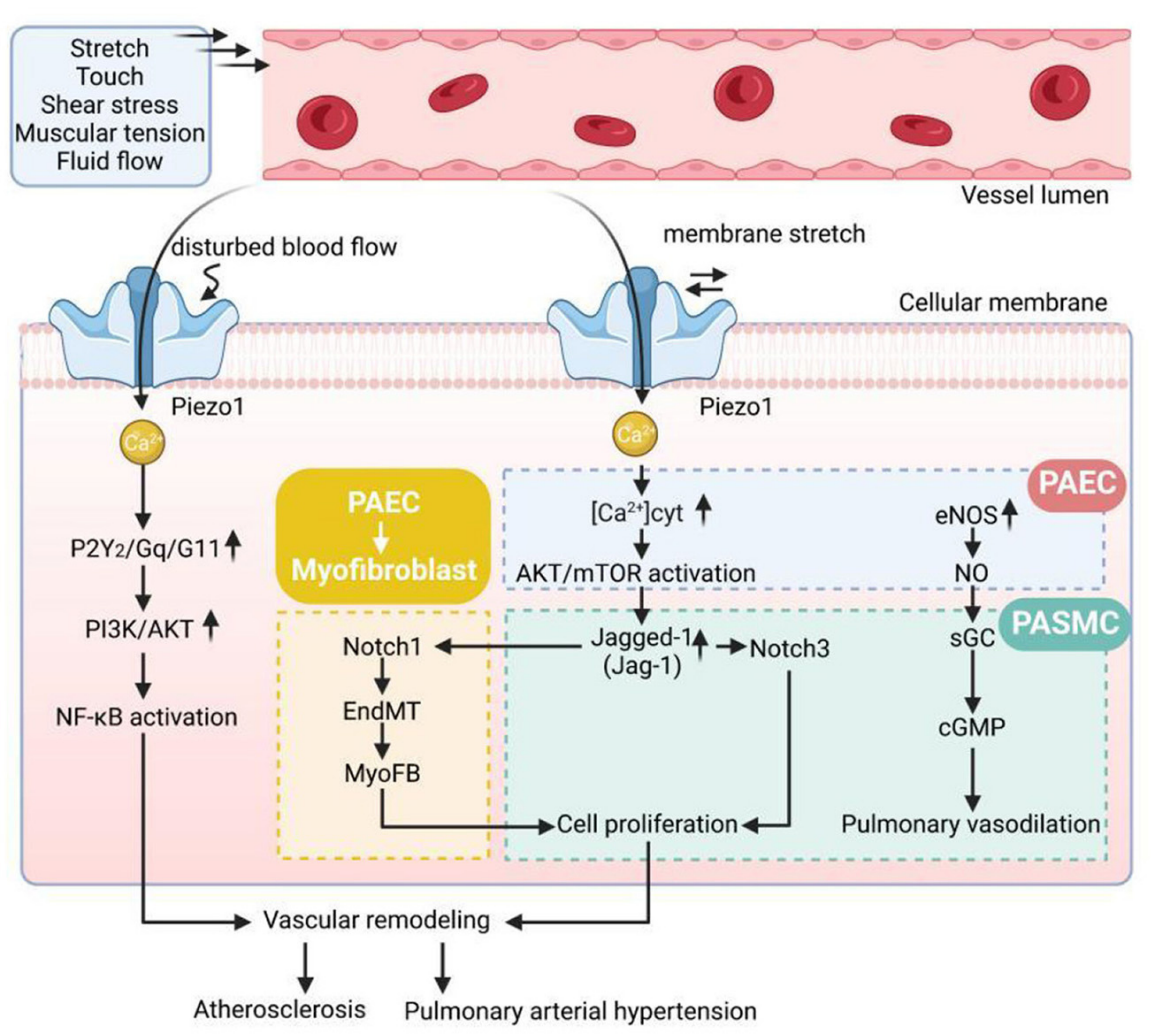
The pathological mechanisms of vascular remodeling disease. Gq/G11, Gprotein alpha-subunits Galphaq/Galpha11; AKT, protein kinase B; PI3K, phosphatidylinositol 3 kinase; NF-κB, nuclear factor kappa B; EndMT, endothelial-to-mesenchymal transition; [Ca^2+^]_cyt_, cytoplasmic free Ca^2+^ concentration; eNOS, endothelial nitric oxide synthase; NO, endothelial nitric oxide; sGC, soluble guanylate cyclase; cGMP, cyclic guanosine monophosphate.

Fernandez et al. (94) found that Piezo1-mediated increases in [Ca^2+^] i were associated with contraction and abnormal proliferation of PASMCs. A dose-dependent relationship with vasoconstriction was found after stimulation of endothelium-denuded rat intrapulmonary arteries by applying Yoda1, and Piezo1 activity was significantly upregulated and increased in idiopathic PAH-PASMCs compared with donor PASMCs, which also explains the effect of proliferation caused by elevated Piezo1-mediated [Ca^2+^] i in PASMCs under PAH on pulmonary artery of the molecular mechanisms of vascular remodeling. In conclusion, these studies further elucidate the role of Piezo1 in vascular remodeling in pulmonary arterial hypertension, which has the potential to be an effective intervention molecule and potential target of action in the treatment of pulmonary hypertension in the future (Figure 1).

## Conclusions

Cardiopulmonary diseases, e.g., hypertension, atherosclerosis, pulmonary hypertension, asthma and chronic obstructive pulmonary disease, are among the most common causes of death worldwide. A common pathophysiological theme to these diseases is vascular remodeling, which is contributed by changes in expression and activation of piezo1 ion channels critical for either excitability or growth. In recent years, many scholars have made remarkable achievements in this field, and we have initially understood that it plays an important role in coupling various signaling pathways to promote vascular development and maintain vascular homeostasis by sensing blood flow shear stress and non-selectively mediating the flow of cations such as Na^+^ and Ca^2+^.

However, our investigation of the role of Piezo1 in vascular remodeling is still in its infancy, and further studies are needed to clarify whether Piezo1 and other mechanosensitive ion channels and membrane proteins act together, and the regulation of upstream and downstream specific signaling pathways when Piezo1 channels act. We believe that with further research on the mechanosensitive ion channel Piezo1, Piezo1 is likely to become a new target for the diagnosis and treatment of vascular remodeling diseases such as hypertension, atherosclerosis and pulmonary hypertension, opening up new ideas for the clinical treatment of vascular remodeling diseases.

## Author contributions

HX, YH, TH, CB, MX, and JL contributed to conception and design of the study. HX and YH wrote the first draft of the manuscript. HX, TH, CB, MX, and JL wrote sections of the manuscript. All authors contributed to manuscript revision, read, and approved the submitted version.

## Funding

This research was supported by the grant from National Natural Science Foundation of China (82174196 and 81770453), Major basic research projects of Natural Science Foundation of Shandong Province (ZR2020ZD16), Youth Natural Science Foundation of Shandong Province (ZR2020QH340), and Traditional Chinese Medicine Development Science and Technology Foundation of Shandong Province (2019-0051).

## Conflict of interest

The authors declare that the research was conducted in the absence of any commercial or financial relationships that could be construed as a potential conflict of interest.

## Publisher's note

All claims expressed in this article are solely those of the authors and do not necessarily represent those of their affiliated organizations, or those of the publisher, the editors and the reviewers. Any product that may be evaluated in this article, or claim that may be made by its manufacturer, is not guaranteed or endorsed by the publisher.
